# Microbial mediated remediation of heavy metals toxicity: mechanisms and future prospects

**DOI:** 10.3389/fpls.2024.1420408

**Published:** 2024-07-19

**Authors:** Haiying Tang, Guohong Xiang, Wen Xiao, Zeliang Yang, Baoyi Zhao

**Affiliations:** ^1^ School of Agriculture and Biotechnology, Hunan University of Humanities, Science and Technology, Loudi, China; ^2^ Shuangfeng Agriculture and Rural Bureau, Loudi, Hunan, China

**Keywords:** bio-sorption, genetic engineering, heavy metals, bioremediation, nano-particles

## Abstract

Heavy metal pollution has become a serious concern across the globe due to their persistent nature, higher toxicity, and recalcitrance. These toxic metals threaten the stability of the environment and the health of all living beings. Heavy metals also enter the human food chain by eating contaminated foods and cause toxic effects on human health. Thus, remediation of HMs polluted soils is mandatory and it needs to be addressed at higher priority. The use of microbes is considered as a promising approach to combat the adverse impacts of HMs. Microbes aided in the restoration of deteriorated environments to their natural condition, with long-term environmental effects. Microbial remediation prevents the leaching and mobilization of HMs and they also make the extraction of HMs simple. Therefore, in this context recent technological advancement allowed to use of bioremediation as an imperative approach to remediate polluted soils. Microbes use different mechanisms including bio-sorption, bioaccumulation, bioleaching, bio-transformation, bio-volatilization and bio-mineralization to mitigate toxic the effects of HMs. Thus, keeping in the view toxic HMs here in this review explores the role of bacteria, fungi and algae in bioremediation of polluted soils. This review also discusses the various approaches that can be used to improve the efficiency of microbes to remediate HMs polluted soils. It also highlights different research gaps that must be solved in future study programs to improve bioremediation efficency.

## Introduction

The world’s population is continuously growing up with a corresponding increase in food demands (Yaashikaa and Kumar, 2022). The recent increase in industrialization and anthropogenic activities are a serious threat to crop production owing to the fact they negatively soil fertility and productivity (Yaashikaa and Kumar, 2022). Various industries excrete toxic heavy metals (HMs) that enter into the soil and negatively affect soil fertility, microbial activities, and crop productivity and these HMs also induce serious effects on humans (**Table 1**) by eating the contaminated foods (Asyakina et al., 2021; Debonne et al., 2021; Nizamutdinov et al., 2022). Global agricultural communities have serious concerns about contamination of agricultural soils with HMs. These HMs are very toxic and they can persist in the soils over a long time period. Different HMs including cadmium (Cd), lead (Pb), zinc (Zn) and copper (Cu) enter into agricultural soils with organic and inorganic fertilizers, while arsenic (As) and mercury (Hg) enter into agricultural soils through nearby located industrial enterprises (Uchimiya et al., 2020; Guan et al., 2022).

**Table 1:** Toxic effects of different heavy metals on human health.

Heavy metals	Toxic form	Health risks	References
Cadmium	Cd^2+^	Cd toxicity reduce cell vitality, induce apoptosis, and damage the kidney, liver and bones.	Wang et al. (2021)
Cadmium	Cd^2+^	High intake of Cd fractured the bones, kidney damage and liver infections along with reproductive dysfunctions.	Kim et al. (2019)
Arsenic	As	As toxicity developed dermal lesions (hyperkeatosis and pigment alterations) and lead to skin cancer,	Muzaffar et al. (2023)
Mercury	Hg	Hg toxicity enhanced heart rate, headache, hypertension, insomnia, alters nerve response, and impairs cognitive function and resulted in cardiac and renal dysfunctions	Eneh et al. (2023)
Lead	Pb	Pb toxicity is lethal to heart, kidney and nervous system. It also affect brain development and gastrointestinal tract of children.	Mishra et al. (2022)
Iron	Fe	Iron toxicity caused dehydrated condition that further develop abdominal pain, Vomiting, diarrhea and lethargy.	Singh et al. (2023)
Cooper	Cu	Cu toxicity caused gastrointestinal distress followed by abdominal pain, vomiting, and hypotension and it also affected the human brain, liver and kidney performances.	Leal et al. (2023)
Chromium	Cr^3+^	Cr^3+^ toxicity reduced cell vigor and cause breast and liver cancer.	Chandra et al. (2020)
Aluminium	Al^3+^	Al damaged central nervous system, kidney and liver dysfunction, and cause pulmonary fibrosis, osteomalacia and lung infections.	Obani et al. (2023)
Vanadium	V	Vanadium toxicity caused nausea and throat injury, rashes and blacken the teeth and tongue.	Briffa et al. (2020)
Mercury	Hg	Hg toxicity disturbed nervous, digestive, and immune systems and dysfunction the lungs, kidneys, skin, and eyes.	Demarco et al. (2023)
Lead	Pb	Pb toxicity damaged fetus brain and kidney along with circulatory and nervous system.	DeOliveira et al. (2023)

Heavy metals are known to accumulate in plants and they negatively affect the plant’s physiological and biochemical processes and consequently cause serious yield losses (Yan et al., 2020). HMs reduce seed germination by negatively affecting the germination related processes which in turn reduce the overall stand establishment (Hassan et al., 2019). HMs also disturb the plant water status, membrane stability and increase the losses of important osmolytes through excessive production of malondialdehyde (MDA) and hydrogen peroxide (H_2_O_2_). Further, HMs also induce excessive reactive oxygen species (ROS) production which damages the proteins, lipids and DNA (Hassan et al., 2013).

Globally, different chemical, physical and biological methods are being used to remove the HMs from soils. Physical methods like thermal treatments, soil washing, vitri-fication, and chemical methods like the application of lime, organic amendments and phosphate compounds are being used to treat the HMs polluted soils (Gong et al., 2018). The physical and chemical methods are quick and efficient; however, they have major limitations. For instance, they are expensive and laborious and they can cause drastic changes in soil quality therefore, these methods offer no optimal solution to treat HMs polluted soils (Gong et al., 2018). Thus, in this context, biological methods offer an alternative solution owing to environmental friendly nature and they are less expensive. The biological methods involve the use of plants (phytoremediation) and microorganisms (bioremediation) to treat the HMs polluted soils (Khalid et al., 2017). The biological methods are economical and environment friendly, and they have appreciable applicability and efficiency as compared to physical and chemical methods (Yan et al., 2020). However, these methods also have some limitations like lengthy periods, environmental sensitivity, and contaminant toxicity (Liu et al., 2023). The use of microbes (bioremediation) got a great scientific attraction across the globe in recent times. The microbes remove the HMs from soil through different mechanisms including bio-sorption, bio-accumulation, bio-volatilization, bio-mineralization, oxidation and reduction, bio-leaching and production of bio-surfactants (Rahman and Singh, 2020). Micro-organism can protect from the negative effects of HMs; however, many HMs destroy membranes of microbial cells. Thus, the ability of microbes to survive under the effect of HMs is an area of decisive importance (Ayangbenro and Babalola, 2017). It has been reported that HMs toxicity and mobility is depend on the degree of oxidation of HMs (Haque et al., 2022). Microbe use HMs pollutants as a food source and change their redox potential (Faskhutdinova et al., 2021). Under HMs stress, some microbes also secrete different substances including polysaccharides, proteins, and lipids that can bind HMs ions and therefore reduce their availability (Martis et al., 2021).

Microbes also reduce the concentration of HMs in soil; for instance, *Aspergillus niger* showed an appreciable ability to for bioaccumulation of Cd and Cr (Khan et al., 2019), similarly, *Stenotrophomonas rhizophila* also significantly removed Pb and Cu by 76.9% and 83.4% (Sun et al., 2021). Due to small size microbes also provide a large surface area to adsorb the HMs which reduces the overall availability of HMs (Rajput et al., 2022). Further, microbes also accelerate the bio-adsorption of toxic HMs which makes them an excellent amendment to remediate HMs contaminated soils (Srivastav et al., 2018). Microbes can also multiply quickly; thus, the use of microbes could be an important amendment to treat the HMs polluted soils (Singh et al., 2020). The recent advancement in microbial bioremediation techniques has shown promising results to remediate polluted soils. For instance, different bioinformatics are being used to develop more effective remediation technologies. These tools are using different databases to explore the underlying mechanisms of degradation (Zheng et al., 2018). Recently, bio-remediation also used genomics, transcriptomics, metabolomics, and proteomics which is added to the evaluation processes of *in-situ* bio-remediation (Villegas-Plazas et al., 2019). Moreover, genomic studies have also allowed us to analyze the genetic information of microbes within the cell which ensures to development of better microbes for remediation (Hakeem et al., 2020). Additionally, recent advancements in synthetic biology also showed promising results and genetically modified organisms (GMOs) have shown appreciable results in removing pesticides, and xenobiotics from the environment (Bala et al., 2022). There are many reviews available regarding the role of microbes in remediating metals polluted soils. Nonetheless, there is no comprehensive review available describing the role of microbes in remediating the antimony, arsenic, cadmium, chromium, mercury, lead, and nickel-contaminated soils. The aforementioned metals/metalloids are highly toxic and their concentration is rapidly increasing in the environment. Recently, bio-remediation got appreciable attention across the globe, therefore, we have discussed the role and mechanisms of microbes to remediate soils polluted by these toxic metals. The current review also discusses the different research gaps that must be filled besides the appreciable progress in the field of bio-remediation. This review provides insights to boost microbial functioning for the remediation of polluted soils.

## Sources of heavy metals entry into soils

Recent industrialization is meeting population food demands but also posing a severe hazard to the environment by excreting poisonous compounds such as HMs (Aluko et al., 2021). These toxic HMs enter into the human food chain by eating the contaminated foods (Sayyed et al., 2019). Among HMs, As, Cr, Cd, Pb and Hg got a serious attention across the globe because their concentrations in many terrestrial, marine, and aerial systems exceed the safety threshold (WHO 1990; Rahman and Singh, 2019). HMs have both natural and anthropogenic origins and they can be found in the atmosphere, water, soil and biological organisms (Yin et al., 2021). HMs generation from human sources is permanent and constant while the generation of HMs from natural sources is also affected by natural sources (Armah et al., 2014). The major human sources of HMs are agriculture, industries and urbanization (Li et al., 2021). Textiles, tanneries, fertilizers, galvanizing factories, metallurgic factories, varnishes, pharmaceuticals and pesticide companies are major sources of HMs pollution (Verasoundarapandian et al., 2022).

In the mining process, a significant amount of waste rocks is produced which contains a low quantity of HMs. These HMs are carried into ground and water areas by biological and chemical leaching and are then enters into the human food chain (Li and Yu, 2015). Agriculture activities also add a significant amount of HMs into soil owing to the continuous use of inorganic chemicals. Natural phosphate contains impurities in the form of HMs, and different HMs such as As, Cd, Ni, Cr, and Zn have been identified in higher concentrations in over 200 phosphate fertilizers used worldwide (Nziguheba and Smolders, 2008). Likewise, pesticides also contained impurities in the form of HMs and it has been found that different pesticides contained Hg, As, Cu and Pb as an active elements. Different pesticides containing Hg (II) and Pb(II) has been banned owing to their higher toxicity (Kothe et al., 2010). The application of industrial and municipal wastewater is also common practice and the constant application of these waste waters also leads to the accumulation of HMs in soil (Ren et al., 2015; Li et al., 2017b). Electronic waste also has a significant contribution in HMs pollution. For instance, in China in electronic waste recycling site has a significant amount of Cd and Cu greater than the threshold levels (Wu et al., 2015). Heavy metals from natural sources include mineral deposition, eruption of volcanic pathogenic processes, and oceanic evaporation (Zhang et al., 2012). Mining is an important source of HMs release (Acosta et al., 2011), and in China mining is produces around 12 lakh ha of wasteland per year with an annual increase of approximately 47,000 ha (Zhuang et al., 2009).

## Effects of HMs on agro-ecosystem

Soil biology is crucial for maintaining soil quality, which is critical for agricultural sustainability. Human activities are a major source of HMs and they disturb soil microbes, soil fertility, and productivity (Sharma et al., 2017). The survival of microbes is negatively corrected with prolonged exposure of HMs like Pb (Yuan et al., 2015). Similarly, coal mining activities also cause a decrease in microbial abundance, biomass, and variability (Nayak et al., 2015). Heavy metals also slow down the breakdown of litter resulting in uneven deposition of litter on the soil (Marschner and Kalbitz, 2003). Furthermore, HMs have a deleterious impact on the breakdown of stream litter (Hogsden and Harding, 2012; Ferreira et al., 2016). Moreover, HMs also induce a negative effect on soil microbes and a negative correlation has been reported between the concentration of HMs and microbial respiration (Nwuche and Ugoji, 2008). Depending on the soil parameters, substrate concentration, and HM exposure, heavy metals can either accelerate or inhibit N mineralization. The toxicity of HMs also disrupts the N transformation pathways which consequently affect the mineralization of HMs (Hamsa et al., 2017). Further, HM pollution also induces a negative effect on N mineralization and nitrification and both these processes decrease with increasing the amount of HMs pollutants. Further, nitrification is considered to be more susceptible to HMs as compared to mineralization (Bewley and Stotzky, 1983). Moreover, HMs also affect the soil enzymatic activities and microbial abundance (Xian et al., 2015) and it has been found that HMs reduce the soil enzymatic and microbial activities and soil microbial abundance (Pan and Yu, 2011; Xian et al., 2015).

For instance, Li et al. (2020) documented that HMs reduced bioactivity, richness, and microbial diversity. They found that heavy metals (Cu, Cr, Ni, Pb, Zn, and Mn) showed total variations of 87.7%, 56.6%, 83.0%, and 55.1% α-diversity, and community composition, predicted by PICRUSt. In another study, it was documented that Pb stress altered the bacterial community structure. These authors found that Pb 2.5% and 5% increased *Actinobacteria* abundance by 118.56 and 147.25% while 5% Pb stress Bacteroidota and Myxococcota increased abundance by 280.76 and 138.54%, respectively (Meng et al., 2023). In another study, a significant change in microbial abundance and diversity was observed in Cd-polluted soil. Cadmium toxicity (50 mg kg-1) increased *Bacteroidota* and *Proteobacteria* by 2 and 0.3 folds while Cd toxicity decreased the abundance of *Acidobacteriota*, *Firmicutes*, *Chloroflexi*, *Myxococcota*, and *Gemmatimonadota* by 0.3, 0.5, 1.7, 2.2 and 2.4 folds (Bandara et al., 2022). The studies have documented that long-term exposure to heavy metals negatively affects soil health. For instance, Cheng et al. (2022) long-term Cd toxicity decreased the soil organic matter, nitrogen, phosphorus, and potassium availability. The other group of authors found that long-term As toxicity showed a negative showed a negative impact on soil enzymatic activities and soil properties. They found that As toxicity reduced the urease and dehydrogenase activities and soil nitrogen, SOM and clay were the main factors affecting the soil enzyme activity (Nurzhan et al., 2022). Some studies also reported that microbial species show resilience in response to HMs. For instance, Philippot et al. (2008) found higher resilience of nitrate reduction rates to Hg stress (100 mg kg^-1^). Brandt et al. (2010) noted that soil bacterial communities showed structural and functional resilience to Cd exposure (0, 40, 150, and 500 mg·kg−1). They found that the observed increase in Cu tolerance against higher concentrations of Cu was involved in the phenotypic adaption and selection at the micro-diversity level. HMs-mediated disruption in soil microbial activities also negatively soil properties and microbial activities. For instance, soils contaminated with HMs are associated with insufficient nutrients, organic matter, and water retention capacity (Singh and Kalamdhad, 2011). The increase in toxicity of heavy decreases the microbial abundance and diversity and indirectly affects soil enzyme activities by changing microbial community synthesizing enzymes (Singh and Kalamdhad, 2016). Moreover, heavy metals also inhibit soil enzymatic activities and reduce the mineralization of SOM and nutrient nutrient cycle (Bakshi et al., 2018). Globally, different including physical, synthetic, and natural remediation techniques (*in situ* and *ex-situ*) are used to remediate polluted soils. The use of genetically modified microbes has received appreciable attention to cleanup metal-contaminated soils and improve stress tolerance (Narayanan and Ma, 2023).

## Plant responses to heavy metals

**Figure 1:**
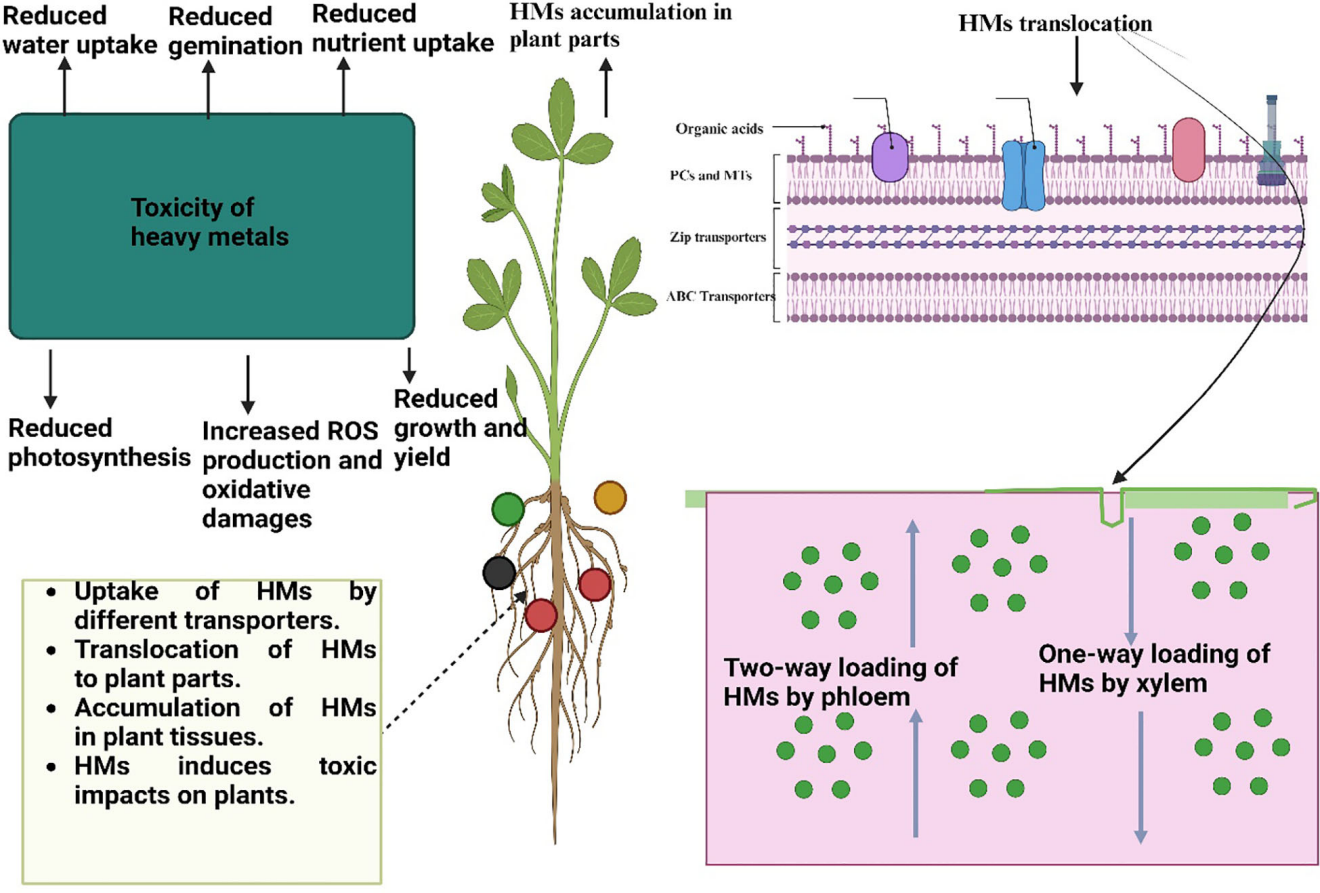
Toxic effects of heavy metals on plants. Heavy metals are absorbed by plants roots and then they are moved to above ground plant by different transporters and then accumulated in above ground parts. The accumulation of HMs in plant parts induce ROS production, necrosis, and decrease nutrient and water uptake and caused protein degradation, cell detoxification thus reduce the plant growth and development.

Heavy metals seriously affect plants and the effects of HMs on plants can be seen from germination to senescence (**Table 2**). Seed germination is one of the most critical stages of plant life and a mediated decrease in seed germination declines seedling growth and subsequent stand establishment (Adrees et al., 2015). For instance, in a study, it was found that combined Cu and Cd stress reduce seed germination, growth of seedlings, and lateral growth rate (Neelima and Reddy, 2003). The exact mechanism through which HMs change seed physiology is not well understood, and different authors reported that HMs inhibit the activities of various enzymes that cause a reduction in seed germination (**Figure 1**). For instance, Hg induced a decrease in seed germination owing to the direct interaction of Hg with HS group proteins that leads to the formation of an S-Hg-S bridge thus causing a loss in enzymatic activities (Cui et al., 2014).

**Table 2:** Toxic effects of different heavy metals on plants and soil health.

Heavy metals	Concentration of heavy metals	Growth media	Plant species	Effect of plants and soil	References
Chromium	120 μM	Soil	Grapevine	Cr toxicity reduced the root and shoot growth, tissue nutrient concentration, chlorophyll contents, leaf water status, quantum yield of photosystem II and soil microbial activity.	Nikolaou et al. (2022)
Lead	200 mg Kg^−1^	Soil	Sunflower	Pb intensity reduced the soil fertility and water uptake along with a significant decrease in stem and root length, dry biomass and crop yield.	Alaboudi et al. (2018).
Cadmium	2 mg kg^−1^	Soil	Rice	Cd stress increased ROS that destroyed the chloroplast and thus reduced the photosynthetic efficiency of plants. Further, Cd toxicity altered nutrient absorption by plant roots.	Li et al. (2023)
Copper	10 g L^-1^	Soil	Barley	Cu toxicity decreased the root and shoot length by affecting stomatal density, conductance and PS II efficiency whereas high Cu reduced the organic matter percentage and microbial population in soil.	Rajput et al. (2018)
Lead	10 mL	Soil	*M. sativa*	Pb toxicity decreased the antioxidant production while increased ROS that reduced the plant growth and physiological functions.	Raklami et al (2021)
Nickle	1000 μM	Soil	Guava	Ni toxicity reduced plant growth and development, photosynthesis and transpiration activities, leaf gas exchanges and K^+^ uptake and microbial growth.	Bazihizina et al. (2015)
Nickle	400 μM	soil	Rice	Ni toxicity reduced the fresh and dry weight along with shoot and root length and increased ROS, lipid peroxidation and consequently protein denaturation.	Hassan et al. (2019)
Lead	100 μM	Soil	Wheat	Pb caused stunted growth, chlorosis and blackening of roots that reduced the soil nutrient uptake mechanism.	Tripathi et al. (2016)
Cadmium	4.8 mM	Soil	Wheat	Nutrient availability reduced to plant under high Cd stress that resulted in decreased root length and seedling growth, subsequently less fresh and dry biomass and yield production.	de Souza Guilherme et al. (2015)
Chromium	300 μM	Soil	Wheat	Cr affected the lamellar system of plant and disturbed the photosynthetic machinery, and caused chlorosis, which impaired growth.	Mathur et al. (2016)

Apart from seed germinations, HMs also change the root architecture and this effect has been reported in plants. In particular, HMs decreased the root elongation (3-4 folds) and enhanced the formation of lateral roots (2-3 folds) in the presence of different HMs like Cu, Pb, Cr, Zn, and Cd (Sofo et al., 2017). The formation of lateral roots is the initial symptom of HMs toxicity which consequently impairs the uptake of nutrients and water thereby reducing subsequent plant growth (Rucińska-Sobkowiak, 2016). Along with root inhibition, HMS also causes a reduction in plant growth. HMs transport from roots to aerial parts and accumulates in plant cells which interfere with cellular metabolism and thus cause a reduction in plant growth (Shanker et al., 2005; Wang et al., 2020). As a result of their interactions with the central atom (Mg) of the porphyrin ring, heavy metals also break down the chlorophyll molecules, severely reducing photosynthesis and ultimately impairing plant growth (Yadav et al., 2014). Moreover, HMs like Cu also cause lignification of both roots and shoots which reduces biomass production owing to impaired cell development (Martins et al., 2020). Additionally, HMs hurt the water relationships which in turn affects a variety of physiological activities like photosynthesis and transpiration (Alsokari and Aldesuquy, 2011). A recent study showed that Cd stress (100 uM) decreased the plant height by 69% and 73% in sorghum cultivars JS-2002 and Chakwal sorghum (Hassan et al., 2019). Further, Cd toxicity also increased MDA concentration by 39% and 43% respectively in both cultivars (Hassan et al., 2019). In another study, it was witnessed that Pb stress decreased the photosynthetic rate, carbon dioxide concentration, transpiration rate, and WUE by 50.5, 73.2, 48.6, and 148.8% respectively (Qin et al., 2023). Heavy metal toxicity also negatively effect nutrient uptake by plants. For instance, *Fava bean* plants’ Cd toxicity (150 mg/L) decreased the Ca and Mg concentration by 1.82 and 1.27 times while Cd toxicity (300 mg/L) decreased the Cd and Mg concentration by 2.278 and 2.25 folds (Piršelová and Ondrušková, 2021).In plants like *Helianthus annuus* and *Vigna radiata*, HMs (As) increased the number of stomata followed by the development of abnormal, arrested, and fused stomata (Gomes et al., 2011; Gupta and Bhatnagar, 2015). Heavy metals also affect the xylem vessels’ parenchymatous and mesophyll cells and resultantly change the plant water relations and are considered to be responsible for the decrease in leaf growth. Heavy metals also negatively affect the photosynthetic machinery however, it depends on the concentration of HMs. Moreover, HMs also negatively affect the light-harvesting, transport of electrons, and RuBisCo activity which in turn reduce the overall plant photosynthetic efficiency (Paunov et al., 2018; Latif et al., 2020). Besides this HMs (Cd) also reduced the photochemical efficiency (Fv/Fm), the effective quantum yield of photosystem II (φPSII), and chlorophyll florescence thereby leading to the inhibition of photosynthesis (Gao et al., 2020; Yotsova et al., 2020).

Generally, HMs (Hg, Cu, Pb, Ni, Cd, and Zn) target the plant chlorophyll in three different ways by increasing the activity of chlorophyllase enzyme, causing oxidation of chlorophyll through increased ROS production, and inhibiting biosynthesis of chlorophyll biosynthesis (Gill et al., 2012; Shahzad et al., 2018; Sharma et al., 2020). HMs not only affect the chlorophyll molecules but also the membranes of the chloroplast and thylakoid cells. For example, swelled thylakoids, degraded chloroplast membranes, and loss of chloroplast membrane were noted in barley plants under Pb stress (Wang et al., 2017). Moreover, HMs also inhibit the light reactions by decreasing the efficiencies of PS-I and PS-II and they also decrease the dark reactions owing to decreased activities of enzymes linked with the Calvin cycle (Souri et al., 2019). Heavy metals also induce overproduction of ROS that damage proteins, DNA, and lipids and lead to the induction of oxidative stress (Foyer and Noctor, 2016). However, plants also activate excellent deference and they also accumulate various osmolytes to counter the toxic effects of HMs. For example, Chowardhara et al. (2020) found that activities of CAT, GST, GR, APX, and POD and accumulation of proline and ascorbic acid were increased in response to Cd toxicity in *B. juncea*. It has been reported that HMs also decrease the uptake of water and nutrient which in turn cause significant growth losses (Rucińska-Sobkowiak, 2016; Wang et al., 2017). For instance, Cd toxicity competes with calcium (Ca), iron (Fe), and magnesium (mg) which caused a significant reduction in growth and biomass production (Raza, 2022).

Under the harmful effects of HMs, nitrogen metabolism is essential for plant growth and development. According to reports, HMs decrease the nitrate and ammonia assimilation enzymes by increasing protease activity. MicroRNAs play an imperative role in HMs toxicity by regulating the plant antioxidant responses, chelations, and auxin and cytokinin signaling (Ding et al., 2020). For instance, Casarrubia et al. (2020) found that mycorrhizal and microRNA played a significant role in Cd tolerance in *Vaccinium myrtillus*. In another study, it was found that MicroRNA expression significantly improved the Cd and Al tolerance in tobacco (Cedillo-Jimenez et al., 2020). Heavy metals also negatively affect the quality of crops and it has been found that Cd toxicity in rice reduced the rice protein contents, and milling degree and increased the kernel chalkiness (Imran et al., 2021).

## Microorganisms responsible for bioremediation

Heavy metal pollution poses a severe threat to public health by contaminating food supplies and drinking water on a global scale (Huang et al., 2020). Microbial remediation is an imperative approach and it has appreciable potential to improve crop productivity, and human health and restore the ecosystem (Narayanan and Ma, 2023). The microbial-mediated bio-accumulation and bio-magnification are very successful in removing the pollutant to ensure safe and sustainable crop production (Manorma et al., 2023). Different microbes including, algae, bacteria, and fungi are being used to clean up the HMs contaminated soils (**Table 3**).

**Table 3:** Different microbes used to remediate heavy metals polluted soils.

Type of microbe	Microbial species name	Used against heavy metals	References
Bacteria	*Penicillium chrysogenum* A15	Lead	Povedano-Priego et al (2017)
Fungi	*A. fumigatus *	Lead	Khan et al (2019)
Bacteria	Pseudomonas sp.	Chromium	Tirry et al (2021)
Fungi	*Penicillium* sp.	Chromium	Barsainya et al (2016)
Bacteria	Phyllobacterium myrsinacearum	Arsenic	Alves et al (2022)
Bacteria	Acinetobacter	Copper	Ke et al (2021)
Yeast	*Wickerhamomyces anomalus*	Chromium	Joutey et al (2015)
Bacteria	*P. fluorescens*	Cadmium	Abbaszadeh-Dahaji et al (2019)
Algae	*Pelvetia canaliculata*	Chromium	Lytras et al (2017)
Bacteria	*PGPE consortium*	Mercury	Ustiatik et al (2022)
Bacteria	*Sinorhizobium Saheli*	Cadmium	Kang et al (2018)
Aerobic bacteria	*Variovorax paradox*	Nickle	Durand et al (2016)
Bacteria	*Bacillus sp. and Bacillus pumilus*	Cadmium	Narayanan and Ma (2023)
Aerobic Bacteria	*Micrococcus luteus*	Arsenic	Pinter et al (2017)

### Bacteria

The interaction of microbes with HMs occurs through different mechanism which depends on metal and microbe type and surrounding environment. Different factors including temperature, pH, nutrient source, and metal ions play an important role in the mobility and bioavailability of HMs for microbial transformation. Bacteria’s small size, rapid growth, and ease of cultivation allow them to thrive in a variety of environmental situations. HMs often connect to functional groups including amino, carboxyl, sulfate, and phosphate groups that are present on the layers of bacterial cell walls (Yue et al., 2015). The potential of bacteria for HMs uptake can vary from 1-500 mg/g. For instance, Hg resistant *pseudomonas aeruginosa* strain absorbed the Hg uptake 180 mg/g (Yin et al., 2016). Likewise, different microbes like *Bacillus* sp. PZ-1 and *Pseudomonas* also absorb the Pb from wastewater (Li et al., 2017a). On the other hand *Arthrobacter viscosus* can absorb the Cr and it also has an excellent capacity to transfer the Cr (VI) into Cr (III) (Hlihor et al., 2017).

*Rhodobacter capsulatus* also showed a maximum capacity of 164 mg/g to absorb the Zn (II) (Magnin et al., 2014) while *Bacillus ceres* showed a maximum bio-sorption capacity of 31.95 mg/g and 24.01 mg for Cd (II) in dead and living cells (Huang et al., 2013). Extracellular polymeric substances (EPS) protect the microorganism from the toxic effects of HMs by restricting entry of HMs into the cell. It has been discovered that EPS has both anion and cationic functional groups, which help to accumulate HM ions like Cd, Hg, Cu, and cobalt (Fang et al., 2017). After adsorption HMs are converted to diverse ionic states in bacterial cells that reduce their toxicity. *Pseudomonas putida*is an important microbe and it can absorb 100% Hg from the marine environment it also reduces the Hg(II) into Hg(0) (Sheng et al., 2018). The findings of Zhang et al. (2012) showed that a new microbial strain *Acinetobacter* sp. showed an excellent ability to detoxify the Cr. In another; authors screened 72 *acidothermophilic*autotrophic microbes for their ability to tolerate and bio-absorb the HMs and these authors found that the ATh-14 strain showed an appreciable potential and it showed absorption capacity of 85.82% for solubilization of copper (Umrania, 2006). Bacteria are better bio-sorbents as compared to other microbes due to their size, ubiquity resilience, and ability to grow under a wide range of conditions (Hlihor et al., 2017).

### Fungi

Fungi also have an excellent ability to remediate the HMs polluted soils. The presence of chitin, polysaccharides, phosphate, and glucuronic acid in fungal cells is essential for the adsorption of HMs (Purchase et al., 2009). Different functional groups and fungal strains had a significant impact on the adsorption rate of HMs (Iram et al., 2015). In a study, it was found that *Termitomyces clypeatus* detoxified the Cr(VI) by adsorbing Cr on its surface through carboxyl, imidazole, hydroxyl, phosphate, and sulfhydryl groups (Ramrakhiani et al., 2011). Further, Amirnia et al. (2015) found that *Saccharomyces cerevisiae* eliminated the Cu(II) from water sources, while Talukdar et al. (2020) found that *Aspergillus flavus* fungal species removed the Cr by more than 70%. Moreover, *Aspergillus fumigates* also showed an appreciable potential to remove the Cd, Cr, Cu, Ni, and Zn from the contaminated soils (Shazia et al., 2013). In another investigation, three different fungal species including *Penicillium citrinum*, *Trichoderma viride*, and *Penicillium* showed a significant potential (250 mg/L) to adsorb the Cr(VI) (Zapana-Huarache et al., 2020).

### Algae

Algae have also shown a good potential to remediate HM-polluted sites owing to the fact algae produce various peptides that help the accumulation of HMs and defend against the HMs (Bilal et al., 2018). For instance, *Fucus vesiculosus* showed a tremendous potential to adsorb the Pb(II) (Demey et al., 2018), likewise, *Cladophora fascicularis* also showed a significant potential to remediate the Pb(II) from wastewater. Similarly, Sargassum marine algae also showed a significant potential to detoxify the Cu (II) from the aqueous solution (Barquilha et al., 2017). In another study Christoforidis et al. (2015) tested the absorption capacity of *Cystoseira crinitophylla* for copper and found that this algae showed a maximum capacity of 160 mg/g to adsorb Cu (Christoforidis et al., 2015). On the other hand, authors noted that *Saccharina fusiforme* and *Saccharina japonica* substantially detoxify the Zn(II), Cd(II), and Cu(II) (Poo et al., 2018) while *Desmodesmus* also showed an appreciable potential to remove the Cu(II) and Ni(II) from the wastewaters (Rugnini et al., 2018). The study findings of Aslam et al. (2019) showed that microalgae showed promising results in the accumulation Mn, Cu, and Zn (Freitas et al., 2011). Moreover, the findings of Freitas et al. (2011) showed that algal biomass showed an appreciable potential for HMs like Fe.

## Factors affecting the bioremediation process

Different factors including metal concentration, valance state, metals bioavailability, redox potential, soil temperature, and pH affect the bioremediation process (Bandowe et al., 2014). The pH of the soil has an impact on bacterial enzymatic activity as well as microbial bio-sorption (Morton-Bermea et al., 2002). Soil pH also changes the surface charge of microbes by affecting the ability of microbes to absorb the HM ions (Galiulin and Galiulina, 2008). Soil pH substantially affects both the transportation and hydration of HM ions in soil (Dermont et al., 2008) and it has been documented that the rate of HMs removal is increased with increasing pH over a certain rate and after this, the rate of removal starts declining (Wierzba, 2015). The ideal pH range for most bacteria is 5.5-6.5 (Wang et al., 2001), however, some bacteria like *Bacillus jeotgali* can thrive at a pH of 7 (Rodríguez-Tirado et al., 2012). Another significant component that influences the absorption of HMs is temperature; which influences the development and proliferation of microorganisms (Fang et al., 2011). Different bacteria require different temperatures to carry out their functions (Acar and Malkoc, 2004). However, HM ions, soil additives, and soil type all have an impact on microbial activity. It is challenging to achieve microbial adsorption due to the low mobility of HM ions caused by soil adsorption and retention of HM ions (Hu et al., 2010).

Soil pH is an important factor that affects microbial growth. For instance, unfavorable pH affects enzyme activity which lowers the rate of microbial metabolism and it also affects the binding capacity between HMs and adsorbants (Bandowe et al., 2014). The changes in pH also affect the mobility and hydration of metals (Bandowe et al., 2014). For instance, the adsorption capacity of Zn and Pb was increased with increasing pH, and an increase in soil pH above 5.5 decreased the removal of Pb and Zn (Wierzba, 2015). Other authors also documented that soil acidification increased the mobility of metals in the following order Cd>Zn>Pb. These authors also document that soil pH affects mobility, causes metal ions to become more or less active, and increases or decreases their environmental risk (Kicińska et al., 2022). Temperature is also a factor that affects microbial growth (Fang et al., 2011). The increase in temperature affects the diffusion of metals and increases the bioavailability of metals. However, optimum degradation temperature can vary according to metal types, for instance, Cd bio-degradation by Bacillus jeotgali was maximum at 35°C while bio-degradation by the same bacteria was higher at 30°C (Chanmugathas and Bollag, 1988). The adsorption efficiency is also affected by soil organic matter, for instance, organic matter tends to fix the metals in soil which reduces the availability to metals (Wang et al., 2022). A short-term study investigates the response of different temperatures (5, 15, and 25oC) Cd, Cu, Pb, and Zn removal by *Carex pseudocyperus*, *C. riparia*, and *Phalaris arundinacea*. Low temperatures reduce the removal capacity of all the metals and an increase in temperature increases the removal capacity of all the metals (Schück and Greger, 2023). Climate change also induces a significant impact on soil microbial activities. For instance, climate-induced variation in soil temperature, and humidity affect the decomposition of SOM and nutrient cycling (Burns et al., 2013), and it partially or fully depends on microbial activity. The change in soil temperature and moisture can change the growth, structure, function, composition, and interaction among microbes for the degradation of pollutants in soils (Alkorta et al., 2017).

Bioremediation is generally limited to bio-degradable compounds, and it is also susceptible to rapid degradation which more toxic compounds. Besides it, bio-remediation also needs extensive monitoring and it has major drawbacks in terms of environmental growth conditions, nutrient requirement, temperature, and pH conditions. Therefore, it is essential to find ways to identify the microbes having a wider adaptability under a wide range of temperature and pH conditions for an efficient remediation process. On a long-term basis, microbial mediation remediation is a simple, cheap, and environmental method and it can improve the overall soil fertility, ecosystem health, and safer and sustainable food production. Nonetheless, implantation of bioremediation needs a comprehensive understanding of soil microbial communities, properties of contaminants, and environmental conditions as these factors play a critical role in getting effective results.

## Microbial mediated remediation of heavy metals polluted soils

**Figure 2:**
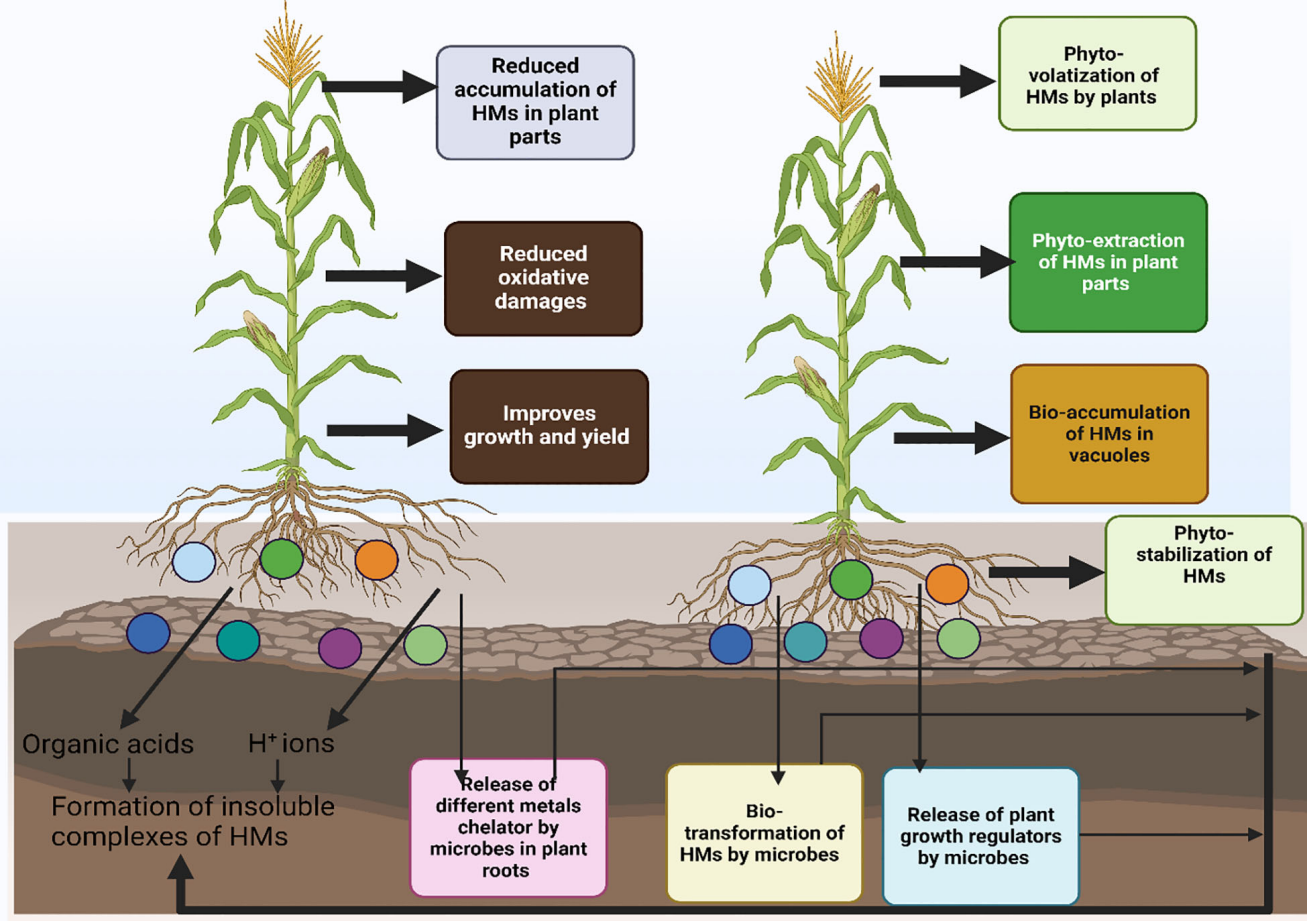
Different mechanism used by microbes to induce heavy metals toxicity in plants. Microbes use different mechanisms bio-sorption, bio-mineralization, bio-accumulation, bio-leaching and bio-transformation to remediate polluted soils. They also increase the availability of nutrients by increasing production of IAA and ACC deaminase, and siderophores thus resulting in better growth under polluted soils.

The use of microbes is considered as an effective way to treat the HMs in polluted soils, as these microbes absorb HMs and also convert them into less toxic forms (Gupta et al., 2016). Microorganisms play a critical role in remediating HMs polluted soils owing to the fact they can with stand metal toxicity. Numerous HMs have been reported to be precipitated, undergo oxidation state changes, and be sequestered by microbes (**Figure 2**; Kang et al., 2016).

## Microbial mediated remediation of antimony contaminated soils

Microorganisms are crucial for remediating Sb polluted soils and they reduce the toxicity of Sb through different ways including, bio-reduction and bio-oxidation (Jeyasundar et al., 2021). Many bacteria have been identified that can be used to remediate the Sb-polluted soils (He et al., 2019). For instance, two bacteria *Shinella and Ensifer* discovered from Sb-contaminated soils showed a tremendous potential to oxidase Sb (Choi et al., 2017) while the bacterial *Bacillales* strain also showed marked results to change the Sb-V into Sb-III (Lai et al., 2018). Similarly, fungi have been also used to remediate the Sb polluted soils, and study findings of Xi et al. (2022) showed that AMF increased plant antioxidant activities by reducing the retention of Sb in plant parts. The findings of Liu et al. (2013) showed that the bacterial strain *Pseudomonas* substantially increased the plant growth, and microbial activity and decreased Sb availability (Liu et al., 2013). In another study Zhang et al. (2012) found that microbes isolated from the rice field contributed significantly towards the oxidation of Sb-III likewise, Li and Yu (2015) also found that *Agrobacterium tumefaciens* contributed towards the oxidation of Sb-III.

Some environmental microorganisms, particularly those that thrive in anaerobic environments, are capable of converting Sb(V) to Sb(III). For instance, Hockmann et al. (2014) noted that microbes converted the SB-V to Sb-III with the help of lactate as an electron donor. Similarly, Kulp et al. (2014) found that microbes in Sb-polluted mines reduced Sb-V to Sb-III. In the case of flooded mine pit soils group of researchers from China found that autotrophic bacteria reduced the Sb-V and generated Sb2O3 by using hydrogen gas (H2) as an electron donor (Lai et al., 2016). Additionally, Huang et al. (2022) found that after 60 days of injection of the *B. cereus* solution into plant roots; the concentration of As and Sb in soil was significantly reduced as compared to soil without bacteria solution this indicates that this strain promoted the absorption of As and Sb from soil (Huang et al., 2022).

## Microbial mediated remediation of arsenic contaminated soils

Arsenic occurs in the environment in different inorganic forms including As-0, As-III, and As-V, and organic forms like dimethylarsinic acid (DMA), monomethylarsonic acid (MMA), trimethylarsine oxide (TMAO) and arsenobetaine. It has been found that bacteria, algae, and fungi can methylate As-III into methylated species (Yang and Rosen, 2016; De Francisco et al., 2021). Different fungal species like *Aspergillus*, *Candida*, *Scopulariopsis*, and *Penicillium* can also cause a change in the methylate inorganic As to the organic As species (Bentley and Chasteen, 2002). It is important to keep in mind is that the ability of certain microorganisms to methylate and volatilize depends on soil organic matter (SOM), soil chemistry, and As concentration (Mestrot et al., 2011). Spagnoletti et al. (2016) tested the impact of AMF (R. *intraradices*) on soybean plants under As stress and found a marked improvement in plant biomass and a reduction in As accumulation. Likewise, Chan et al. (2013) also found that AFM (*Geosporum*) enhanced the phosphorus uptake and reduced the As concentration in rice grains.

Transgenic microbes are also an effective way to treat As toxicity. For instance, transgenic microbes with expressed arsM showed an ability of 2.2-4.5% to remove As from soil while the same microbe showed an ability of 10-fold in nutrient solution (Liu et al., 2011). In another study, Huang et al. (2015) found that thermophilic strain *Bacillus subtilis* 168 was unable to do methylation and volatilization of As. They genetically modified this bacteria with CmarsM gene and found that genetically modified bacteria caused methylation and volatilization of As it occurred within 48 hours in As-contaminated organic compost. Moreover, Villadangos et al. (2014) also modified the As-resistant bacteria named *Corynebacterium glutamicum* by ArsC1 and ArsC2. These authors found that As(V) was significantly increased after the introduction of genetically modified bacteria. Additionally, Preetha et al. (2023) also prepared the mutant *C. glutamicum* strain and found that this strain showed an ability of 15 folds and 30 folds more to accumulate As-III and As-V as compared to the control treatment.

## Microbial mediated remediation of cadmium contaminated soils

Cadmium is a very toxic HM posing a serious threat to human health and the environment. The application of microbes is an effective and promising technique to treat Cd-polluted soils. In a study, Ma et al. (2020) discovered the Cd immobilization PGPR (TZ5) and found that this bacteria significantly increased ryegrass weight by 77.78% and decreased the concentration of Cd in ryegrass by 48.49%. Further, the application of this bacteria also increased the soil enzymatic activities and microbial growth which indicates that this bacterial strain (TZ5) can provide a practical approach to remediate Cd-polluted soils (Ma et al., 2020). Limited studies are conducted to determine the impacts of single and co-inoculation of *Bacillus mycoides* and *Micrococcus roseus* on growth and nutrient uptake of maize grown under Cd stress (100 and 200 mg kg-1). These authors found that all bacterial treatments appreciably improved the plant growth and biomass and the combination of both bacteria reduced the root and shoot Cd uptake and transfer and translocation as compared to control (Malekzadeh et al., 2012).

In Cd-contaminated soils, Cd-tolerant bacteria play an important role (Bravo, 2022). The microbes use various mechanisms including biosorption and intra-cellular accumulation to mitigate the adverse impacts of Cd stress (Ghosh et al., 2022). Recently, genetically modified organisms also played an important role in remediating Cd-polluted soils (Abbas et al., 2018). Different genetically modified organisms (CdtB Enterobacter and Klebsiella variicola) showed an appreciable potential to remediate Cd polluted soils (Feria-Cáceres et al., 2022; Quiroga-Mateus et al., 2022). Similarly, Arce-Inga et al. (2022) found that the application of *Theobroma cacao* (CCN51) significantly decreased the uptake of Cd, and its translocation to plant parts. Feng et al. (2023) studied the impact of *mixotrophic acidophiles* under Cd-contaminated soils. These authors also found that soil solution pH and reduction level of glucose affected the abundance of *Acidithiobacillus* which contributes significantly towards removal of Cd (Feng et al., 2023). On the other hand, the fungal strain belonging to *Purpureocillium lilacinum* tolerated the Cd stress up to 12000 mg/L. The SEM analysis indicated Cd can be accumulated on the mycelial surface generating plenty of metal precipitation particles. Further, these authors also found that in pot experiments this fungal strain also reduced the soil Cd concentration in soil by 12.56% and promoted plant growth this indicates that this fungal could be an important candidate to remediate Cd polluted soils (Deng et al., 2021).

## Microbial mediated remediation of chromium contaminated soils

Chromium is released into the environment as a result of human and anthropogenic activities which pose a serious threat to living organisms. Microbial remediation is an effective approach to treatment the Cr polluted soils. For instance, in a study authors tested the impact of *Nostoc linckia* to remediate Cr polluted soils. They found that this microbe showed an appreciable potential to accumulate Cr and suggested that this bacteria could be an effective candidate to remediate Cr-polluted soils (Cepoi et al., 2021). In another study, edaphic cyanobacteria were tested for Cr remediation and it was found that these bacteria produce polysaccharides, glycoproteins, lipopolysaccharides, and ionic functional groups that can coordinate with Cr and reduce its availability (Cheung and Gu, 2007). Moreover, different Cr-tolerant bacteria including *Bacillus*, *Enterobacter*, *Pseudomonas*, and *Streptomyces*have been identified and they can remove the Cr by 50-90% (Ramesh and Winkler, 2010; Bansal et al., 2019; Elahi and Rehman, 2019; Murthy et al., 2022). The study findings of Wen et al. (2023) showed that the addition of SR-2, PA-1, and LB-5 improved the plant fresh weight by 10.3%, 13.5%, and 14.2% and increased the soil enzymatic (catalase and sucrose) activities and significantly decreased the shoot Cr concentration by 19.2-83.6%.

Chen et al. (2021) studied the impact of *B. cereus* WHX-1 on mitigating Cr toxicity. They found that this microbial species improved the soil physicochemical properties, soil bulk density and decreased the redox potential. They also found that this microbial species transferred the Cr-IV by 94.225 into Cr-III increasing the residual fraction of Cr by 63.38%. Further, these authors also found that the application of *B. cereus* improved the growth and biomass production of ryegrass. In another study, Ahmed (2018) studied the impact of chromium-tolerant auxin-producing rhizobacteria on growth characteristics of *Lens culinaris* growing under different Cr concentrations (0, 50, 100, 200, 400, and 500 µgml−1). The results of their study findings showed that *Bacillus* species mitigated the deleterious impacts of Cr reduced the Cr accumulation in soil and reduced Cr availability to plants.

## Microbial mediated remediation of lead contaminated soils

Bioremediation with microbes is considered an effective approach is a promising technique to remediate the Pb-contaminated soils. For instance, a pot study conducted on wheat showed that *R. sphaeroides*reduce the Pb concentration in root and lead by 14.78% and 24.01% (Li et al., 2016). On the other hand, Rhee et al. (2012) found that two fungal species *Paecilomyces javanicus* and *Metarhizium anisopliae* isolated from mining produced organic acids that resulted in precipitation of Pb. In another research study Sun et al. (2017) found that soil inoculation with *M. circinelloides* significantly increased the Pb removal by *S. nigrum* L. These authors also found that soil fertility was also increased after inoculating the soil with *S. nigrum* (Sun et al., 2017). Likewise, Zhou et al. (2016) added WH16-1 strain in Pb2+ contaminated paddy soil and found that this bacterial strain decreased the exchangeable and carbonate-bound Pb in the paddy soil 14.04 and 10.69% (Zhou et al., 2016).

The study findings of Puyen et al. (2012) showed that *Micrococcus luteus* marked decreased Pb concentration in soil, likewise, findings of Kalita and Joshi (2017) showed that *Pseudomonas aeruginosa* application to Pb-polluted soil appreciably reduction the concentration of Pb with 40 mg g-1 sorption capacity (Kalita and Joshi, 2017). Shanab et al. (2012) tested the potential of different algae to remediate Pb-polluted soils and they found that different algae isolates like *Phormidium ambiguum*, *Pseudochlorococcum typicum*, and *Scenedesmus significantly* reduced the Pb toxicity. Fungi is also an effective candidate for reducing Pb toxicity (Fawzy et al., 2017) application of AMF under Pb stress effectively increased the sunflower biomass and mitigated the toxic effects of Pb (Hassan et al., 2013). In addition to producing various organic acids, polyphosphates, peptides, and sulfur compounds, fungi also do cell wall binding, and make chelate, and precipitate that decreases Pb toxicity (Bellion et al., 2006).

## Microbial mediated remediation of mercury contaminated soils

Mercury microbial remediation needs the microbial species to withstand and remove the Hg over extended periods. Various authors noted that microbes effectively remediate the Hg-contaminated soils. For instance, *Vigna unguiculata* inoculated with *Photobacterium* and grown on Hg-contaminated soil (27 mg/kg) showed increased root growth (11%), seed production (33%), leaf numbers (50%), Hg uptake in roots (25%) and decreased Hg concentration in aerial plant organs (55%) as compared to un-inoculated control (Mathew et al., 2015). Similarly, two bacterial strains like *Brevundimonas diminuta* and *Alcaligenes faecalis* applied to Hg and Pb-contaminated soil increased the phyto-accumulation of Pb and Hg by roots and shoots (Hamzah et al., 2015). In another study Hg resistant microbials including *Enterobacter ludwigii* and *Klebsiella pneumoniae*, promoted plant growth and decreased proline concentration, MDA concentration and electrolyte leakage in wheat seedlings growing under Hg stress 75 μM; (Gontia-Mishra et al., 2016).

In another study, bacteria inoculation significantly improved maize growth and reduced the Hg uptake by maize plants growing under Hg (Mariano et al., 2020). Fungi have also shown an appreciable potential to remediate Hg-contaminated soils and it has been found that AMF inoculation increased the plant growth, P uptake, and reduced Hg uptake as well as translocation in *Lactuca sativa* growing under Hg stress under (10 mg/kg) (Cozzolino et al., 2016). Moreover, commercial AMF like *Glomus*, *Entrophospora* and *Scutellospora genera*, appreciably improved the seedling growth and root elongation of rice plants growing under Hg toxicity (Vargas Aguirre et al., 2018). Another group of authors also found that commercial AMF also promoted plant growth and stimulated the uptake of Hg in *Lolium perenne* and rice plants growing under Hg toxicity (Leudo et al., 2020). Likewise, Pietro-Souza et al. (2020) found that compared with *Chrysopogon zizanioides* plants growing with AMF under Hg stress showed a marked improvement in plant growth, root and shoot biomass, chlorophyll concentration and showed a reduction in Hg accumulation. Moreover, *Aspergillus* and *Curvularia geniculata* also appreciably increased the maize root growth, root dry weight, shoot dry weight, chlorophyll, and Hg accumulation by 40% and 34% respectively (Pietro-Souza et al., 2020).

## Microbial mediated remediation of nickel contaminated soils

Microorganisms are extremely important for the bioremediation of Ni-polluted soils owing to the fact this method is very economically effective against Ni toxicity (Hassan et al., 2019). Various bacterial strains including *Bacillus thuringiensis* and *Bacillus cereus* have shown promising results in treating the Ni contaminated soils (Zhu et al., 2016). Cabello-Conejo et al (2014) recorded that *Arthrobacter nicotinovorans* appreciably improved plant growth and increased the phyto-extraction of Ni from polluted soil. Zaidi et al. (2006) documented that *Bacillus subtilis* decreased the toxicity of nickel while noticeably boosting mustard growth and nickel phyto-extraction. Other authors also found that inoculation with *Trichoderma atroviride*and *Glomus intraradices* improved the Ni phyto-extraction and reduced the Ni toxicity in linseed and mustard (Cao et al., 2008).

In another study, Alboghobeish et al. (2014) tested the potential of bacterial strain (*Klebsiella oxytoca*) and found that this strain showed a Ni tolerance of 24 mM. Likewise, *Enterobacter asburiae* from industrial water depicted the Ni tolerance to a 15 mM concentration and it removed the 75% Ni by bio-accumulation (Paul and Mukherjee, 2016). Heidari and his colleagues found that a *Microbacterium oxydans* strain showed a Ni removal efficiency of 83-91% (Heidari et al., 2020) while Das et al. (2014) reported that *Bacillus thuringiensis* found that removed the Ni by 82% through bio-sorption process. According to Costa and Tavares (2017), Alternaria and Penicillium species have respective Ni biosorption potentials of 11.3 and 13.1 mg g-1. Trichoderma and Aspergillus inoculation also considerably increased the effectiveness of Ni’s phytoextraction (Jiang et al., 2008) and Stenotrophomonas from industrial waste also showed an appreciable potential to remove the Ni (Aslam et al., 2020). However, the biosorption of Ni by microbes significantly affects microbial strain, pH, temperature, and initial Ni concentration (Heidari et al., 2020).

## Microbial resistance to heavy metals and their mechanisms

During HMs stress microbes either die owing to toxicity developed by HMs or they thrive in this condition through different resistance mechanisms against HMs (**Table 4**). Microbes develop different mechanisms including, extra and inter-cellular sequestration, and extracellular barriers, and they actively transport the metal ions to tolerate HMs toxicity. On the surface of bacteria, there are several barriers such as cell walls, plasma membranes, and other structures like EPS that prevent HMs from entering bacterial cells (Bhati et al., 2019). The research findings of Kumar et al. (2014) indicated that bacteria and fungi cause the bio-sorption of metals like Cu, Pb, and Cr. Microbial biofilms contain polymers that accumulate HM ions and protect the inside bacterial cells and the presence of biofilm on *Pseudomonas aeruginosa* showed tolerance against, Cu, Pb, and Zn (Teitzel and Parsek, 2003). Further, the presence of biofilms also increased the elimination efficiency of HMs (Grujic et al., 2017). Additionally, cell walls and EPS also work as an excellent barrier and they substantially adsorb the metal ions like Pb and Cr (Kushwaha et al., 2017).

**Table 4:** Microbial remediation of heavy metals contaminated soils and different mechanism used by microbes to remediate heavy metals contaminated soils.

Type of microbe	Microbial species name	Used against heavy metals	Potential mechanism	References
Bacteria	*Bacillus* sp. KL1	Nickel	Biosorption	Taran et al. (2019)
Algae	*Spirulina sp.*	Chromium	Biosorption	Rezaei (2016)
Bacteria	*Bacillus thuringiensis*	Nickel	Immobilization of Ni	Zhu et al (2016)
Algae	*Spirulina platensis*	Chromium	Biosorption	Kwak et al (2015)
Bacteria	*Streptomyces sp. NRC21696*	Arsenic	Chelation	AL-Huqail and El-Bondkly (2022)
Bacteria	*Sphingomonas paucimobilis*	Chromium	Enzymatic transformation	Ibarrolaza et al (2011)
Yeast	*Candida tropicalis*	Chromium	Biosorption	Bahafid et al. (2013)
Bacteria	*Acidithiobacillus*	Nickel	Bioleaching	Wu et al. (2020)
Bacteria	*Aspergillus spp.*	Nickel	Oxidation and reduction	Bisht and Harsh (2014)
Yeast	*Cyberlindnera fabianii*	Chromium	Biosorption	Fernández et al. (2018)
Bacteria	*Bacillus sp. E1S2*	Cadmium	IAA production and ACC deaminase synthesis	Ma et al. (2015)
Fungus	*Ganoderma lucidum*	Lead	Biosorption	Chang et al. (2020)
Filamentous fungi	*Aspergillus niger*	Chromium	Biotransformation	Gu et al (2015) Singh et al. (2021)
Bacteria	*Aspergillus niger*	Nickel	Biosorption	Oyewole et al (2019)
Fungi	*Phanerochaete chrysosporium BKM-F-1767*	Lead	biosorption and bioaccumulation	Huang et al (2017)
Bacteria	*Bacillus amyloliquefaciens*	Chromium	Biosorption/Bioreduction	Fernández et al (2018)

ACC, 1-Aminocyclopropane-1-carboxylate; IAA, indole-3-acetic acid.

The cellular membranes of microorganisms contain additional proteins and metabolic products that interact with HMs to decrease their availability. Microbes also develop extracellular sequestration which involves the complexation of metal ions as insoluble compounds and this mechanism is an important way to reduce the HMs toxicity (Thelwell et al., 1998). Microbes also develop intra-cellular sequestration in the metal ions form complexes with distinct compounds in the cell cytoplasm and this is a very common mechanism used by microbes to withstand the toxicity of HMs. Microbes with the aid of low molecular proteins like cysteine accumulation HMs like Cu, Cd and Zn intra-cellularly (Higham et al., 1986) and other microbes like *Rhizobium leguminosarum* use glutathione to accumulate HMs (Cd) intra-cellularly (Lima et al., 2006). The cell wall of fungi is made of lipids, chitin, polysaccharide, polyphosphates, and proteins which help them to accumulate HMs both intracellularly and extra-cellularly (Remenar et al., 2018).

Numerous metal exporting proteins, including ABC transporters, P-type efflux ATPase, cation diffusion facilitator, and proton-cation anti-porters, are found in microorganisms and assist in the efflux of harmful metals (Soto et al., 2019). ABC transporters also help microorganisms tolerate the stress brought on by HM by facilitating the ions’ transfer across membranes (Lerebours et al., 2016; Zammit et al., 2016). The microbial resistance to HM is also contributed by enzymes that transfer the HMs ions from hazardous to less toxic forms (Giovanella et al., 2016; Liu et al., 2017). Microbes use different mechanisms including, biotransformation, extrusion, EPS production, and proteins to survive the toxicity of metals (Wu et al., 2009). They also produce different proteins like metallothioneins that bind heavy metals thereby reducing HMs toxicity (Wu et al., 2010). Further, EPS produced by microbes is a mixture of proteins, nucleic acid, and polysaccharides that find metals and reduce their concentration in the surrounding environment. Different mechanisms including electrostatic interaction, ion exchange, precipitation, redox process, and surface complexation are involved in processes (Yang et al., 2015). The enzymes transfer metals into less toxic forms in cells through oxidation, reduction, complexation, sequestration, methylation, and de-methylation. Different enzymes like arsenite oxidase, mercuric reductase, chromate reductase, and nickel-coenzyme m reductase have been identified to convert the metals Lerebours et al., 2016; Zammit et al., 2016).

## Microbial mechanism used clean up HMs polluted soils

Different mechanisms were used by microbes to clean up the HM-polluted soils. Microbes play a critical role in the oxidation of metals, for instance, *Thiobacillus ferrooxidans*) can promote the oxidation of metal sulfides to enhance the release of HMs. Microbes mediate the transformation of metal sulfides by sulfur oxidation. In this process, microbes oxidize sulfide ores into metal ions by the process of biological leaching (Kaksonen et al., 2020). Microbes also cause the reduction of metals to reduce their toxicity. The removal capacity of HM-nFeS against Cr-VI was 12-20% lowest as compared to DM-nFeS which was linked with the capacity of both HM-nFeS and DM-nFeS to reduce the Cr (Du et al., 2016). The details of various mechanisms used by microbes to remediate the HMs polluted soils are discussed below.

## Bioaccumulation and biosorption

Bioaccumulation and biosorption are the most common mechanisms used by microbes to remediate polluted soils and in both mechanisms, microbes bound the HMs from the surrounding environment (Joutey et al., 2015). In bio-sorption microbes use cellular structure to capture the HM ions and then absorb these HMs on the binding sites of cell walls (Malik, 2004). Microbes also used adsorption mechanisms as bioremediation of HM. Different microbes including *Magnetospirillum gryphiswaldense*, *Bacillus subtilis*, microalgae, *Chaetomorphalinum*, *Rhizopus arrhizus*, and *Saccharomyces cerevisiae* produce biosorbents for remediation of HM (Zhou et al., 2012). In comparison to other microbes, bacteria are thought to be superior bio-sorbents because of their larger surface-to-volume ratio and variety of chemosorption sites in their cell walls, including teichoic acid (Beveridge, 1989). Dead bacterial strains also have good biosorbent properties and it has been found that dead *Bacillus sphaericus* showed 13-20% more bio-sorption capacity for Cr as compared to living cell of the same strain (Velásquez and Dussan, 2009). On the other hand, bio-accumulation depends on an import storage mechanism. This process is known as active bio-accumulation and it involves the movement of HM ions across the lipid bilayer of the cell membrane and into the cytoplasm or intracellular regions. The bioaccumulation of HM in bacterial membranes is mediated via a variety of ionic channels, carrier-mediated transports, permeation, and lipid permeation (Shahpiri and Mohammadzadeh, 2018). In literature, it has been well documented that microbes cause bioaccumulation of Pb, Ni, Hg, Cd, and Cr (Rani and Goel, 2009; Sher and Rehman, 2019; Naskar et al., 2020). Different researchers also identified the micro-bacterium that shows resistance to HMs. For instance, Henson et al. (2015) reported that Microbacterium sp. (Cr-K29) reduced the Cr-IV uptake by 88% while Pattanapipitpaisal et al. (2001) found that Microbacterium liquefaciens eliminated the Cr by 90-95%. These microbes use heavy metal ions in order to facilitate their metabolic activities or they also use enzymes produced by bacterial cells to detoxify ions of HMs (Kubrak et al., 2010).

## Bioleaching

Bioleaching is another important mechanism used by a wide range of microbes to remediate polluted soils. For instance, in a research study, authors found that *Acidophiles* and *chemolithotrophs* oxidized the Fe-II to Fe-III and reduced sulfur to sulfuric acid. The production of sulfuric acid leads to the synthesis of ferric ions as well as protons which helps to extract metals through solubilizing oxides and sulfides of metal (Srichandan et al., 2014). Microbes are utilized in bioleaching as reduction agents, but they can also be used to extract and recover HMs (Wang and Zhao, 2009). Bio-remediation has been offered as an excellent tool to recover raw materials from effluents (Gadd, 2010). Using an Annona squamosa-based absorbent with 0.1 M HCl, Isaac and Sivakumar (2013) achieved Cd recovery efficiency of 98.7%. Contrarily, matrix-immobilized P. putida cells demonstrated 100% recovery for Cu while Pseudomonas aeruginosa biomass demonstrated 82% recovery efficiency for Cd (Hammaini et al., 2007). In another study, co-application *Pseudomonas aeruginosa* biomass, and hydrochloric acid (0.1 M HCl) achieved the Cd recovery rate by 82% (Dickerhof et al., 2019), while*P. putida* achieved a Cu recovery rate of 100%. Further, autochthonous variant *Enterobacter* brought an exceeding recovery of >90% for Cu and Pb (Bayramoglu and Arica, 2011). Acidphiles produce different acids through their metabolic process which aids in the dissolution of metal ores, thereby reducing the availability of metals. On the other hand, chemolithotrophs cause oxidation and reduction of sulfur compounds which provide energy to them and also increase the production of acids subsequently increasing solubilization of metals. The bio-leaching carried by both acidophiles and chemolithotrophs is eco-friendly and it can be carried at lower temperatures along with additional benefits of energy saving (Adetunji et al., 2023).

## Biotransformation

In biotransformation, microbes converted the toxic metal ions to less hazardous forms (Pervaiz et al., 2013). To adapt to environmental changes, bacteria have developed bio-transformation mechanisms. Production of carbon bonds, isomerization, functional groups, oxidation, reduction, condensation, hydrolysis, methylation, and demethylation help the microbes to transform the HMs. These are all processes that can be used to alter HM in microbes. Microbes cause the transformation of HM and Nagvenkar and Ramaiah (2010) noted that *Micrococcus*and *Acinetobacter* caused the oxidation of As-III into less soluble and non-toxic form. Moreover, Thatoi et al. (2014) documented that Cr (VI) tolerant *Bacillus*species cause the biotransformation of Cr (VI) and changed it into a less hazardous form of Cr (III). Both Micrococcus and Acinetobacter reduce the toxicity of metals by causing oxidation, reduction, biological chelation, and inducing the metabolic transformation and bio-film formations (Adetunji et al., 2023).

## Bio-volatilization

Bio-volatilization is a process where microbes convert the HMs into volatile compounds enzymatically. This process significantly reduced the availability and toxicity of metals in soil and water. Bio-volatilization uses enzymatic reduction and methylation to convert toxic metals into less toxic forms. Different enzymes like Arsenic methyltransferase, Mercury reductase, and Antimony methyltransferase are involved in the bio-volatilization of As, Hg, and Sb. This method is considered to be suitable for HMs like Hg, As, and Sb, and in this process, these HMs are converted into non-toxic compounds by bio-volatilization (Boriová et al., 2014). Bacterial enzymes like *methyltransferases* transfer the As(V) into the mono, di, and tri-methylated As species which is then transferred into the atmosphere owing to its volatile nature. In another study, enzymes like reductase (MerA) and mercurial lyase present in archaea and eubacteria caused bio-volatilization (Freedman et al., 2012). Similarly, *Scopulariopsis brevicaulis*, also showed promising results to convert the As(V) and Hg(II) to their nontoxic states (Urík et al., 2007; Boriová et al., 2014).

## Bio-mineralization

In the bio-mineralization process, microbes activate the synthesis of minerals and microbes to tackle with HMs. Different bacteria cause immobilization of Pb and Cr by carbon mineralization (He et al., 2019). Similarly, another bacterial strain *Sporosarcina ginsengisoli* caused immobilization of different HM calcite, aragonite, and vaterite biomineralization (Achal et al., 2012; Cheng and Holman, 2012). Fungal species also showed promising results for bio-mineralization, for example, *Penicillium chrysogenum* causes mineralization of Pb and Cr (Qian et al., 2017). Likewise, *Penicillium chrysogenum* effectively causes bio-mineralization of Pb (Povedano-Priego et al., 2017) additionally, due to the synthesis of PO43- that is released during the breakdown of Pb, Bacillus subtilis triggered bio-mineralization of Pb (Lin et al., 2016). Moreover, other authors reported that *Pseudomonas putida* forms the carbonate and phosphate minerals which speed up Cd precipitation (Li et al., 2016). Microbes play a critical role in the bio-mineralization process as this process involves the production of mineral deposits to immobilize HMs. The microbes produce EPS, specific metabolites, and organic acids which promote the formation of mineral deposits thereby leading to the immobilization of HMs (Qian et al., 2017). The siderophores and polysaccharides produced by microbes bind the HMs by forming complexes with metals thereby reducing uptake and accumulation of metals by plants. Besides this, they also facilitate the sequestration of metals in soil thereby reducing toxicity of metals on plants.

## Modern approaches used to remediate HMs contaminated soils

Different techniques are being applied globally to clean up HM-polluted soils. The role of modern approaches to remediate HM-contaminated soils is discussed below.

### Phyto-microbial system for remediation of polluted soils

The application of plants and microbes has emerged as an excellent tool to remediate HM-polluted soils. The use of PGPR has been tested as an effective, and environmentally friendly way to eliminate HMs (Sati et al., 2023). Different microbes like bacteria and fungi can help the plants absorb the HMs (Bojórquez et al., 2016). For instance, Joner and Leyval (1997) noted that fungal inoculated plants uptake more Cd by 90, 127, and 131% growing under different Cd levels (1, 10, and 100 mg/kg) as compared to un-inoculated plants. Similarly, fungal inoculation improves the plant’s ability to absorb Cu, Cd, and Zn (Sati et al., 2023). Different PGPB also produce polysaccharides which increase the transformation, immobilization, and chelation of HM thus reducing their availability. PGPB decreases soil pH by increasing the production of organic acids which helps to remove the HM ions, further, these PGPB also provide nutrients to plants thus reducing the negative effects of HM on plants. Siderophore is also an important microbe and it has shown an appreciable ability to form complexes with different metals like Al, Cd, Cu, Zn, and Pb (Rajkumar et al., 2010). When bio-augmentation and phytoremediation are used together, they produce noticeable results and can also get around some of the challenges that arise with using them alone. The plant also showed significant results to remediate polluted soils and according to Wang et al. (2021), planting Salix in soils with Cd contamination increased the diversity of helpful fungi and microorganisms and contributed to impressive bioremediation outcomes. Plant growth-promoting rhizobacteria (PGPR) interact with plants to increase their ability to absorb HMs through a different mechanism like the production of chelators, increased nutrient uptake, volatilization, transformation, and phytostabilization. This technique is considered sustainable and eco-friendly which can help to mitigate the HMs pollution in agricultural settings.

### Genetically engineered microbes: key player to remediated HM polluted soils

The recent advancements in genetic engineering and the production of genetically modified microbes have shown promising results for the remediation of polluted soils. Molecular biology involves understanding and changing the genes to improve the bio-remediation process. It has been documented that different microbes possess resistance mechanisms against HMs (Jaiswal et al., 2019). This includes genes that encode different metal proteins, transporters, and enzymes involved the detoxification (Malla et al., 2018). Thus, engineering these genes can allow for an increase in the microbial ability to effectively carry the microbial remediation process. The recent advance in CRISPR-Cas9 also ensured the editing of microbial genes and the introduction of new genes resulting in improved performance of microbes against HMs (Lee and Lee, 2021). The microbial metabolic pathways can also be modified which can enhance the microbe’s ability, while fine-tuning genes is also leading to better remediation capabilities. Moreover, omics and microbial consortia engineering also provided insights into the response of microbes to HMs (Peña-Castro et al., 2023). This can help to identify different genes, regulatory pathways, and elements to improve the remediation process (Peña-Castro et al., 2023).

The literature shows that genetically modified microbes have a better capacity to remove the HMs (Bhatt et al., 2022). The editing of a single gene and changing the sequence of the gene are important practices used to produce genetically modified microbes (Diep et al., 2018). Different HMs like Cd, Cu, Hg, Ni, and Fe are eliminated by engineered bacteria (Azad et al., 2014) however, the degradation rate largely depends on enzymes present in bacterial cells (Kang, 2014). Moreover, the use of recombinant DNA technology and the introduction of foreign genes has also allowed to develop the genetically modified microbes. For instance, the use of genetically modified *Pseudomonas putida* and *Escherichia coli*e effectively removed the Hg from polluted soils (Deckwer et al., 2004), similarly, the addition of mer operon from *Escherichia coli* to bacterium *Deinococcus geothemalis* also reduce the Hg pollution even at higher temperature (Dixit et al., 2015).

*Cupriavidus metallidurans* modified genetically with pTP6 plasmid also significantly reduced the Hg from polluted soils (Dixit et al., 2015). The use pMR68 plasmid to introduce novel genes into Pseudomonas also led to the development of Hg resistance (Sone et al., 2013). To enhance the bioremediation of HM, microbial membrane transporters can also be genetically engineered and in this context, transporters and binding mechanisms play a critical role to remediate polluted soils (Manoj et al., 2020). When HMs enter the cell, several phytochelatins, metallothioneins, and polyphosphates collaborate to sequester the HM and alter the HM key storage system, enhancing their ability to take HMs from soil and water (Diep et al., 2018).

The use of genetically modified microbes (GEMs) has speeded up the remediation process. For the successful implementation of implementation of GEMs bacteria must be capable of tolerating the antagonism induced by other native bacterial species (Dixit et al., 2015). Therefore, more novel approaches to screening as well as isolation of microbes for remediation of polluted soils must be used. Recently, different approaches like genomics, metagenomics, metabolomics, proteomics transcriptomics, and computational biology have been used to develop the GEMs for the remediation of HMs (Raza et al., 2024). The recent advancement in high throughput techniques has allowed us to identify the genes involved in the bio-remediation of diverse metals. Further, recent techniques like CRISPR-Cas also made it possible to create GEMs containing genes that can break down the HMs. Besides this, it also made it easy to transfer the desired set of information into microbial genomes to develop the microbes with better ability (Miglani, 2017).

CRISPR-Cas9 techniques have also allowed to development of microbes with appreciable precision, high efficiency, and targeting multiple metals. Genetically modified microbes can provide better results to remediate polluted soils. For instance, genetically modified microbes enhance metal uptake capacity, and they have better metal tolerance and resistance with minimal environmental impacts. They also have appreciable sequestration, transformation, detoxification, and uptake abilities which make them effective tools to mitigate metals toxicity. However, many potential ethical and environmental implications must be considered when using genetically modified microbes for the remediation of polluted soils. For instance, it includes proper regulation and monitoring of genetically modified organisms to balance the benefits of reduction in contamination along with potential risks. Other concerns could be human health, environmental quality, and the negative effects of farming practices. Many environmental considerations must be used while using genetically modified microbes. These microbes should not disrupt biodiversity, food webs, and ecosystem health.

### Use of nano-technology for microbial remediation of HM polluted soils

Nano-materials have documented appreciable results in remediating polluted soils owing to their higher surface area, reactivity, and surface chemistry (Khati et al., 2017; Baragaño et al., 2020. Different types of nano-materials including zero-valent metals, metal oxide nanoparticles, carbon-based nano-materials, nano-composites, and nano-biosensors are used around the globe to remediate polluted soils (Aliyari et al., 2023). The nano-materials serve as electron donors in the microbial reduction process and they promote the reduction of toxic metals. On the other hand, nano-particles serve as absorbents and they also favor HMs degradation (Dhanapal et al., 2024). Further, carbon-based produces also enhance the transfer of electrons among metal ions and microbes which in turn increases the efficiency of bio-remediation. Recently, nano-biosensors have also shown appreciable results in detecting HMS which has allowed the monitoring of the remediation process (Dhanapal et al., 2024). Nano-biosorbents can be employed as a substitute for conventional bio-sorbents (Alviz-Gazitua et al., 2019). There are various functional groups found in NPs, including NH2, -COOH, and -OH, and customizing the right functional groups by activating them physically or chemically or by altering their surfaces has produced promising results for the elimination of HMs. Additionally, bacterial strains produce the NPs that can aid in the bio-remediation of the HMs (Arshad et al., 2019). It has been shown that using NPs in conjunction with microorganisms boosted the reduction of HMs, producing more beneficial benefits than using them alone. Nano-particles have a higher surface area, ion exchange, reduction and stabilization capacity, mobility, and delivery which enhance the remediation efficacy. The interaction between NPs and microorganisms is, however, influenced by a variety of factors, including NPs’ chemical properties, size and shape, coating qualities, crystalline phase, level of contamination, and resistance to hazardous elements (Tan et al., 2018). Their tailored properties and enhanced adsorption capacities make them promising candidates for sustainable and efficient remediation strategies, provided that environmental and safety considerations are carefully addressed in their application. However, nano-sorbents must be tested for their environmental impacts in terms of stability and NPs release into the environment. Microbes trapped with nano-materials produce the nano-composite, a combination of *Halomonas*and iron oxide NPs substantially eliminated the Cd-II and Pb-II (Cao et al., 2020). Since, separation and recovery of HMs from nano-materials is laborious and time-consuming thus magnetic NPs gained significant attention in recent times, wherein surface amendment, coating of diverse materials, and encapsulation focused on simple separation of HMs.

## Conclusion and future research directions

Heavy metals pollution is a serious issue across the globe and it is considered the biggest challenge of this century. Heavy metal pollution has drastic effects on soil quality, soil fertility, microbial activities, and diversity, and it also impost deleterious impacts on human health by entering the food chain. Globally, different physiochemical strategies are used to remediate the HMs polluted soils. However, these strategies are very expensive, difficult to application, inefficient in certain conditions and they can also alter the soil quality. Therefore, new biological methods have been developed to remediate polluted soils. Among biological methods, the use of microbes is considered as an effective, economical, and eco-feasible measure to remediate polluted soils. The microbes use different mechanisms to remediate polluted soils and recently engineered microbes provided excellent results for bioremediation which makes them an effective measure to be used on polluted soils.

The use of a single strategy could be both noneffective and inefficient in reclaiming polluted soils. Therefore, a combination of microbes and plants, nano-particles, and additives could also be an important approach to remediate polluted soil. Moreover, a combination of microbes with other strategies including organic and carbon-based materials must also be tested. Additionally, to create the HM tolerance in microbes more focus must be done to understand the physiochemical, biological, and molecular characteristics of microorganisms in soil and water habitats where HMs are prevalent. In the literature, no studies are available about the long-term effects of altering soil pH, temperature, and redox conditions for bioremediation efforts on soil health, microbial diversity, and the persistence of heavy metals over a long period. Therefore, efforts must be made to study the long-term impacts of soil pH, temperature, and redox conditions on on soil health, microbial diversity, and the persistence of heavy metals. Besides this, there is also a lack of information about the interactions between metal concentration, pH, redox potential, and temperature affecting the efficiency and effectiveness of microbial bioremediation processes in contaminated soils. Thus, it is interesting to study the interactions between metal concentration, pH, redox potential, and temperature affecting the effectiveness of the remediation process.

To identify prospective metal resistance and detoxification genes that can be regulated in other species to improve their particular performance, meta-genomic techniques, and microbial metabolic studies are required. Additionally, genetic study is required to comprehend the routes and mechanisms that plants and microorganisms use to tolerate and detoxify heavy metals. The recent advance in omics-based approach can also help to develop the strains tolerant against the prevalent environmental conditions. Recently, yeast has been modified and it showed promising hyper-accumulation capacity, therefore, other bacteria can also be developed in the same way to clean the polluted soils. Future research should pay more attention to the usage of algae since it may be a promising strategy for the sorption of heavy metals. The application of nanotechnology in combination with microbes can also promote microbial use and their efficiency on polluted soil.

## Author contributions

HT: Writing – original draft. GX: Writing – original draft, Writing – review & editing. WX: Writing – review & editing. ZY: Writing – review & editing. BZ: Writing – review & editing.
